# Effects of acetaminophen on hepatic microcirculation in mice

**DOI:** 10.1186/1476-5926-2-S1-S33

**Published:** 2004-01-14

**Authors:** Yoshiya Ito, Nancy W Machen, Edward R Abril, Robert S McCuskey

**Affiliations:** 1 Department of Cell Biology and Anatomy, College of Medicine, University of Arizona, Tucson, AZ, 85724-5044, USA

## Introduction

Acetaminophen (APAP) intoxication from overdosing can result in severe hepatic damage, which is characterized by hemorrhagic centrilobular necrosis and by towering the levels of transaminase. The APAP-induced hepatic necrosis is preceded by centrilobular microvascular congestion thought to be due to collapse of the sinusoidal wall and the infiltration of blood elements into the space of Disse [1]. These findings suggest that, in addition to direct hepatocellular damage, sinusoidal endothelial cells (SECs) participate in liver injury elicited by APAP overdose. As a result, the present study was conducted to examine changes in hepatic microcirculation after APAP administration using *in vivo *microscopic methods.

## Methods

APAP (600 mg/kg) was given to male C57Bl/6 mice by oral gavage. At 0, 0.5, 1, 2, 4, 6, 12 h after APAP, the hepatic microvascular responses in anesthetized animals were examined using established high resolution *in vivo *microscopic methods [2]. The relative adequacy of blood perfusion through the sinusoids was evaluated by counting the number of sinusoids containing blood flow (SCF) in ten periportal (PP) and ten centrilobular (CL) regions in each animal. To examine the interaction of leukocytes with the sinusoidal wall, the number of leukocytes adhering to the endothelial lining of sinusoids was counted. Endothelial swelling was assessed by counting the numbers of swollen cells. Kupffer cell phagocytic activity (KC/SCF) was assessed by counting the number of Kupffer cells (KC) that phagocytosed injected fluorescent latex particles in relation to the number of SCF in the same microscopic fields. To quantify the extent of hemorrhage elicited by APAP gavage, the area occupied with extra-sinusoidal red blood cells (RBCs) was measured using a computer-assisted digital imaging processor. The results were expressed as extra-sinusoidal area occupied with RBCs (micrometer^2^/10 CL regions). In some animals, to assess the functional integrity of the endothelium, formaldehyde-treated serum albumin (FSA) labeled with rhodamine isothiocyanate (RITC) (gift of Dr. B. Smedsr–d, University of Troms–), which is a specific ligand for the scavenger receptors unique to the hepatic SEC [3], was injected. RITC-FSA was visualized by epi-illumination with filter combination. In a separate set of experimental animals, blood was collected from the inferior vena cava. The serum activities of alanine aminotransferase (ALT) and tumor necrosis factor (TNF) alpha were measured by enzymatic procedures and by bioassay, respectively. In addition, the amounts of inducible nitric oxide synthase (iNOS) mRNA in the liver tissue were determined by using semi-quantitative RT-PCR (Quantikine mRNA, R&D System Inc., Minneapolis, MN). All data were expressed as means – SEM. Multiple comparisons were performed using one way analysis of variance (ANOVA). Differences were considered to be significant for p values less than 0.05.

## Results

**Figure 1:**
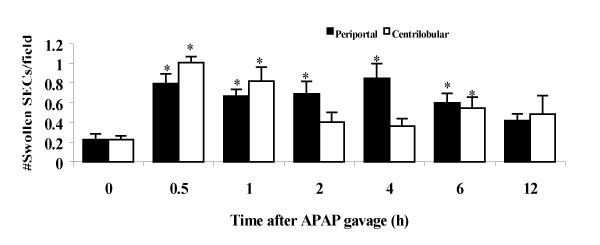
Changes in the numbers of swollen sinusoidal endothelial cells (SEC) after APAP gavage. *p &lt; 0.05 vs. untreated controls (time 0).

**Figure 2:**
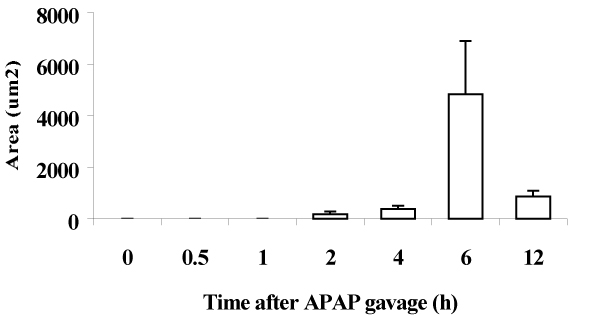
Changes in the extra-sinusoidal area occupied by red blood cells (RBCs) after APAP gavage.

The levels of ALT increased minimally up to 2 h; thereafter they were significantly and progressively increased (from 980 IU/L 4 h to 6300 IU/L 12 h). The numbers of SCF in CL regions were significantly and progressively decreased, and reached a nadir level at 6 h after APAP (by 38% decrease). APAP caused no significant change in the numbers of leukocytes adhering to the sinusoids in both PP and CL regions. The numbers of swollen SECs in PP regions were significantly increased 2.7- to 3.8-fold from 0.5 to 6 h after APAP (Fig. 1). These numbers in CL regions were peaked (4.5-fold) at 0.5 h after, and also were significantly increased (3.6-fold) at 1 h after, again (2.4-fold) at 6 h APAP treatment. Erythrocytes infiltrated into the space of Disse in CL regions as early as 2 h, and the area occupied with these cells was markedly increased at 6 h. (Fig. 2). At 2 h after APAP, the *in vivo *staining for FSA in centrilobular SECs appeared to be weak. With time after APAP dosing, the FSA-staining became faint, and 12 h after APAP, the staining was no longer detectable in CL sinusoidal linings. APAP significantly increased phagocytic Kupffer cell activity in PP regions (1.2- to 1.5-fold) from 0.5 through 12 h after APAP. In contrast, the phagocytic activity of Kupffer cells in CL regions was significantly elevated at 2 h (2.3-fold) and 4 h (1.8-fold) after APAP, and again at 12 h (2.3-fold) after APAP. The levels of TNF-alpha bioactivity were significantly elevated (6.6-fold) and peaked at 4 h after APAP, and declined thereafter. The amount of iNOS mRNA significantly increased 3.1-fold within 1 h after APAP, and peaked at 6 h after (19.6-fold).

## Discussion

The results of the present study demonstrate early events occurring in the hepatic microvasculature following oral gavage with a toxic dose (600 mg/kg) of APAP, notably the swelling of SECs and activated phagocytic Kupffer cell function. At 0.5 and 1 h after APAP, numerous swollen SECs were observed. Two hours after APAP gavage, erythrocytes penetrated into the extra-sinusoidal space, as evidenced by extra-sinusoidal area occupied with RBCs in centrilobular regions, while no significant increases in serum ALT were shown. The functional integrity of SECs was impaired in terms of uptake of FSA. Parallel to the rising the hepatic transaminase levels, the extra-sinusoidal area progressively increased to maximal levels 6 h after APAP gavage. It remains unclear about the mechanisms by which the formation of gaps in SEC after APAP administration, however, depletion of GSH levels in SEC *in vitro *might be attributed to alterations in cytoskeleton of SEC receiving APAP [4]. The involvement of Kupffer cells in APAP hepatotoxicity has been shown [5]. In the present study, Kupffer cell phagocytic activity was elevated during the development of APAP-induced liver injury. We also showed that TNF-alpha levels were significantly increased from 4 h after APAP dosing, suggesting that TNF-alpha could contribute to APAP-induced liver injury [6]. However, TNF-alpha may not be involved in the initiation of SEC injury elicited by APAP, because TNF-alpha levels were increased later than the onset of SEC injury. In this regard, free radicals including NO have been suggested to be an important mediator of APAP hepatotoxicity [7]. Indeed, hepatic iNOS mRNA was detectable within 1 h after APAP and peaked 6 h after APAP. Thus, NO may promote APAP-induced hepatocellular damage as well as SEC injury due to the formation of peroxynitrite [8]. In conclusion, the present study suggested that SECs were a direct and early target for APAP intoxication, and that SEC injury could contribute to the initiation or progression of APAP-induced liver injury.
